# Multifocal Cellulitis due to Disseminated Neisseria Gonorrhoeae in a Male Patient

**DOI:** 10.14740/jocmr1732w

**Published:** 2014-03-31

**Authors:** Yusuke Yoshino, Minami Abe, Kazunori Seo, Ichiro Koga, Takatoshi Kitazawa, Yasuo Ota

**Affiliations:** a Department of Internal Medicine, Teikyo University School of Medicine, 2-11-1 Kaga, Itabashi-ku, Tokyo 173-8606, Japan

**Keywords:** Neisseria gonorrhoeae, Disseminated gonococcal infection, Cellulitis

## Abstract

We report a rare case of disseminated gonococcal infection in a 37-year-old man presenting with multifocal cellulitis. The patient presented with fever and painful swelling of the right foot and left hand, and was admitted to our hospital. CT scanning of the extremities revealed multifocal cellulitis. Transthoracic echocardiography findings were normal, and piperacilin/tazoactam therapy was initiated. On antibiotic day 4, *Neisseria gonorrhoeae* was cultured from a purulent effusion collected from a focal site. *Chlamydia trachomatis* was detected in urine samples by PCR. We made the diagnosis of multifocal cellulitis due to *N. gonorrhoeae* in a patient with chlamydia urethritis. The antibiotic agent was changed from piperacilin/tazobactam to ceftriaxone. Levofloxacin was also administered for chlamydia urethritis. By admission day 14, all lesions had resolved and administration of antibiotic agents was terminated. Disseminated gonococcal infection, although rare, should be included in the differential diagnosis of all sexually active patients who present with multifocal cellulitis - also a rare condition, particularly in light of the fact that in recent times, patterns of sexual activity have changed, which was a pertinent factor in this case.

## Introduction

*Neisseria gonorrhoeae* are gram-negative cocci that can cause sexually transmitted disease, including urethritis, epididymitis or cervicitis. The usual symptoms of focal infection in men are burning on urination and penile discharge. Women, on the other hand, are asymptomatic half the time, or have vaginal discharge and pelvic pain.

*N. gonorrhoeae* also can cause systemic infection. At first, *N. gonorrhoeae* infects locally. Subsequently, it increases in the focal site, and then spreads systemically. Disseminated gonococcal infection (DGI) results from bacteremic spread of *N. gonorrhoeae*, which can lead to a variety of clinical symptoms and signs. The classical triad of features consists of dermatitis, tenosynovitis and migratory polyarthritis [1]. Endocarditis and meningitis also can occur, but these are extremely rare and have been reported only as case reports. DGI is quite rare, occurring in 0.5 to 3% of cases [2]. Gender is an important risk factor, in that DGI is four-fold more common among women than men because local symptoms in males are more severe than those in females, and so men tend to be more aware of their infection [3]. Here, we report a rare case of DGI in 37-year-old man presenting as multifocal cellulitis.

## Case Report

**Figure 1:**
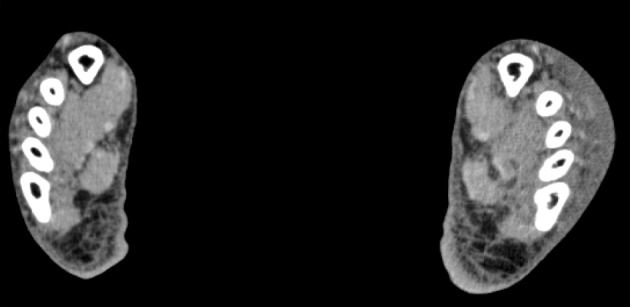
The CT scan of the lower extremities revealed edematous changes in adipose tissue of the upper side of the left foot.

The patient presented complaining of fever, throat pain and painful swelling of the right foot and left hand for 1 week, and was admitted to our hospital. Past medical history included a diagnosis of liver cirrhosis (LC) due to alcoholic fatty liver at the age of 36; the patient has continued to drink. He often used the services of sex workers and had a history of gonococcal urethritis at the age of 34. One month prior to admission, he had unprotected sexual contact. On admission, physical examination revealed a temperature of 39.0 °C, pulse rate 124 bpm and blood pressure 140/76 mmHg. There were painful, reddish and swollen areas on the top of the right foot and the back of the left hand. On the right foot, purulent effusion that was leaking from a small scar was collected for culturing. Blood was also collected for culturing. Admission laboratory findings included a white blood cell count of 13,500/μL with a left shift and a C-reactive protein level of 27.80 mg/dL. A CT scan of the extremities revealed cellulitis in the right foot and left hand (Fig. 1).

Multifocal cellulitis was diagnosed and piperacilin/tazoactam (4.5 g every 6 h) therapy was initiated. Transthoracic echocardiography was performed to search for the focus of this infection, but there were no abnormal findings. On admission day 4, the purulent effusion cultures revealed an *N. gonorrhoeae* strain sensitive to third-generation cephalosporins. We made the diagnosis of multifocal cellulitis due to *N. gonorrhoeae*, and we considered it to be a manifestation of DGI. Piperacilin/tazobactam was changed to ceftriaxone (2 g every 24 h). A throat swab obtained 4 days after initiation of antibiotic therapy remained negative for *N. gonorrhoeae*. On admission day 9, *Chlamydia trachomatis* was detected in urine samples by PCR, and oral levofloxacin (500 mg every 24 h) was begun. After initiation of antibiotic agents, all symptoms, including the fever and painful swelling of the extremities, gradually declined. After switching antibiotic agents, symptoms continued to decline. On admission day 14, all symptoms had resolved and administration of antibiotic agents was terminated.

## Discussion

According to a few previous reports, the incidence of DGI has recently increased [4, 5], particularly among males, partially because changes in sexual behavior have resulted in the fact that sites other than the genital organs, such as the pharynx, can become reservoirs for *N. gonorrhoeae* [5]. In our case, although *N. gonorrhoeae* was not detected from the throat swab collected 4 days after initiation of antibiotic therapy, the pharynx might have been harboring the bacteria, because throat pain was reported one week before admission. Therefore, this case might illustrate typical findings given new trends discussed in recent reports.

In our case, *N. gonorrhoeae* was cultured from the purulent effusion, but not from other sites. *N. gonorrhoeae* is difficult to culture due to its fragility. Our diagnosis of DGI was made because *N. gonorrhoeae* was cultured from focal sites and the patient responded to antibiotic treatment, with resolution of all symptoms. Molecular biological testing, including nucleic acid amplification testing, should be performed on samples from patients suspected to have *N. gonorrhoeae* infection who have negative culture results.

Skin and soft tissue infections are unusual presentations of DGI, but there have been several case reports since 1926 [6-8]; although these might be overlooked as rare presentations of DGI, there may be an increase in cases with diverse presenting symptoms in the future because of changes in patterns of sexual activity. For example, it was already reported that an increase in the number of men having sex with men is related to the increase of rectal gonoccocal infection [9]. In addition, the number of immunocompromised patients (like our case, with chronic liver disease) is increasing because of treatment improvements, a fact that also may increase the incidence of DGI and lead to changes in presenting symptoms.

In conclusion, we have reported a rare case of DGI in a male patient having multifocal cellulitis as the presenting symptom. Although definitely rare, cases of DGI presenting with various symptoms will be increasing in the future, perhaps especially in the male population. Our case illustrates the fact that DGI should be included in the differential diagnosis for all sexually active patients who present with multifocal cellulitis.
